# Surgical Miscellanies

**Published:** 1843-04

**Authors:** 


### Art. VIII.

1. *Heelkundige Mengelingen.* Door J. F. Kerst, Chirurgiæ Doctor, &c.—*Utrecht*, 1835. 8vo, pp. 256. Met Platen.

*Surgical Miscellanies.* By J. F. Kerst, Doctor of Surgery, &c.—*Utrecht*, 1835. With Plates.

2. *Klinische Bijdragen tot de Theorie en Praktijk der Genees en Heelkunde.* Door C. Gobee, Med. en Chir. Doctor.—*Utrecht*, 1839. 8vo, pp. 320. Met twaalf Tabellen en eene Plaat.

*Clinical Contributions to the Theory and Practice of Medicine and Surgery.* By C. Gobee, Doctor of Medicine and Surgery.—*Utrecht*, 1839. With twelve tables and a plate.

Although these works have been published some years, they were unknown to us until very recently; and we doubt not they will be new to nearly all our readers in this country. Dutch is so little read by foreigners that authors writing in that language are more likely to be overlooked than the writers of any other nation, save by the small circle of their countrymen. For this reason we have endeavoured, to the best of our power, to make known to our readers the most esteemed works that have reached us from Holland; and it will always be a gratification to us to be able to prove to the eminent men of that country how highly we appreciate their honorable character, and their important labours in the field of medical science. On the present occasion, we think that the best purpose, for both authors and readers, to which we can use the works now before us, consisting chiefly of miscellaneous observations, is to extract those parts from them which possess the most interest.

I. The first chapter of Dr. Kerst's book is occupied by a report of the most important events in the Royal Military Hospital of Instruction at Utrecht during 1830, and parts of 1831 and 1834. He describes (p. 5) a preparation of a *remarkable case of necrosis of the sternum*, from an adult man. The whole manubrium has perished, and in its place there is a sac of the same form, but larger. The walls of the sac are chiefly membranous, as if formed by the persistent periosteum, but they have several isolated plates of bone, tough and newly formed, adhering to their internal surface: the posterior wall is flat, the anterior is arched and perforated by six apertures. The sac contains three loose, spongy sequestra. The cartilages of the upper ribs are firmly attached to its sides, and its walls seemed to be continued into the periosteum of the clavicle, the fibrous membrane thus prolonged being all that remains in the place of the sterno-clavicular articulation.

A case is related (p. 29) of *excision of the external malleolus.* The patient was a soldier, twenty-two years old, in whom chronic ulceration of the lower extremity of the fibula ensued in consequence of a sprain. The disease had existed fourteen months, and his health was much impaired. The skin over the outer ancle was thick, hard, and painful, and there was a constant slight discharge from an aperture which led into the substance of the fibula. Having cleared the malleolus from the soft parts over it and by its sides, Dr. K. tried to work a small chain-saw between the fibula and tibia; but, as there was not room enough for this, he employed a trephine, and cut out a piece of the fibula. He then opened the joint, and by raising the bone as much as possible with a spatula, he divided all the ligaments and completed the removal of the diseased portion. Severe inflammation of the parts ensued soon after the operation, and when this was subdued abscesses formed in the sole of the foot; but, at length, a cure was established, and the patient left the hospital with a useful though partially anchylosed joint.

To this succeeds a case (p. 35) of removal of the os cuboides, with the last two metatarsal bones and toes, which were all carious. In three months the patient had recovered, with perfect mobility of all the remaining joints, and could walk very well without a stick.

The next case (p. 40) is one of a lad seventeen years old, who, two years previously, fractured his skull to such an extent that ten applications of the trephine were thought necessary. A portion of his left parietal bone, about four inches square, was thus removed; but he quickly recovered, with the exception of a paralysis of the right side, and this also gradually disappeared. Many observations were made upon the state of his brain, which was covered only by the cicatrix of skin and dura mater. Respiration seemed to have no influence at all upon it. Pressure on the cicatrix, to the full extent to which it would yield, produced no effect. But, which is most remarkable, the centre of the cicatrix where the bone was deficient, was always level with the surrounding surface of the skin in the morning, but in the course of the day it gradually fell to about an inch (Rhenish) below that level, and rose again in the course of the night. The patient himself had no consciousness of this change.

As an appendix to the first chapter, Dr. Kerst adds some "brief notes." One relates to a patient who had always enjoyed good health, till inflammation, with ulceration of the ancle-joint, occurred in consequence of a sprain, and required the removal of the leg. While the stump was healing, a comrade, bidding him "good bye," shook him heartily by the hand, and, slight as the force was, it brought on immediately an acute inflammation of the wrist, with disease of the carpal bones, and, in spite of all treatment, the poor fellow had to lose his fore-arm. The stump healed very well by the second intention, but in less than a year he died of phthisis, with caries of the upper dorsal vertebræ. Another brief note tells of a soldier who suffered from disease of the hip-joint, and in whom sloughing from pressure in lying destroyed the whole coccyx: yet, when he recovered, he was as well able as ever to discharge and retain his fæces.

The second chapter comprises a report of what occurred in the surgical department of the military hospital at Antwerp, during the bombardment in September and October, 1830. It is almost exclusively occupied with gunshot wounds, and contains its full share of marvellous courses, flattenings and splittings of bullets, and the other singularities of such accidents. Among them a case is mentioned in which a shot passed through the head of the tibia without splitting it, and the patient did well, without amputation. Others are related which make it probable that longitudinal fissures of bones occur only when the balls strike obliquely, and that they always follow the same direction as that in which the ball was moving.

The following are the most remarkable of the cases related in this chapter. A man received a gun-shot wound which exposed two inches of the right parietal bone. There was no appearance of fracture, but he had severe headach: on the following morning he became comatose, and soon died. On examination, the outer table of the bone was found entire, and was not depressed: the inner table was fractured to the same extent as the outer was exposed, and the broken portions were pressed deeply in upon the brain, and had excited acute inflammation of its membranes.

A soldier had his hand shattered by the accidental discharge of a gun loaded only with powder. He instantly fell senseless; but in four hours he came to himself, and had great pain in the arm with inability to move it. In two hours more the whole limb became insensible, and one of his fingers was removed without his feeling it. There were no signs of affection of the brain, except a dilatation of the left pupil. The paralysis of the arm continued complete for a long time; and the author ascribes it to concussion of the brachial plexus.

Four amputations at the shoulder-joint were performed by La Faye's method slightly modified, and all were successful. The thigh was amputated at the hip-joint twice. The first patient was excessively reduced by long-standing necrosis of the femur, and died three hours after the operation. The second had a gun-shot wound; secondary amputation high up was being performed, when the bone was found to be splintered nearly as far as the joint. Incisions were therefore carried up from the circular incisions already made, and the head of the bone was removed. This patient, like the first, died as if from the shock of the operation, which he survived only forty-eight hours.

The following points in Dr. Kerst's treatment of gun-shot wounds, which in general is very simple and judicious, deserve notice. He is very favorable to the employment of general bleeding, whenever robust patients have not lost much blood from their wounds; and after the general bleeding, but not unless it has first been employed, he uses local bloodletting. In wounds implicating important organs he abstracts very large quantities of blood; in many cases, as much as from 50 to 100 ounces in a few days from the arm, and whatever 300 or 400 leeches will draw from the wounded part; his rule being, to go on with the bleeding, as long as the pulse is above its natural strength, and till the usual period of inflammation is past.

With respect to amputations, he found the rules laid down by English surgeons, to be generally correct, though he managed to save some limbs which, had he strictly acted up to those rules, he must have condemned. It is to be remembered, however, that he was practising in a town, not in the field, nor in a moving army. Perhaps to the same circumstance may be referred the fact, that his secondary amputations had more favorable results than his primary ones. Of 36 amputations of large members, 16 were performed within the first twenty-four hours, and 20 at later periods; of the former he lost 8 or one half (two by hospital-gangrene); of the latter, only 4 or one fifth, and among these 20 were the four amputations at the shoulder-joint.

The treatment after operations was of the simplest kind. He began his practice at Antwerp, by trying in all cases to obtain union by the first intention; but he found that the attempt was so rarely successful and so often productive of inconvenience, that he soon gave it up and adopted a kind of middle course which proved much more advantageous. He covered the wound with linen, spread with some simple ointment, and a small quantity of soft charpie, then, with strips of plaster, brought its edges within about an inch of each other, and applied a layer of lint and a bandage upon the stump. This dressing was repeated till the wound was healed, or else, if all seemed going on well, the edges were brought together on the 3d or 4th day, and allowed to unite. In amputations of the thigh, the stump was always carefully bandaged, to prevent the too great retraction of the muscles; and only two cases of conical stumps occurred.

In the third chapter of the work, which contains a report of the chief events during Dr. Kerst's service in the field, there are but few cases of interest. The most remarkable is one of *dislocation of the humerus upwards and backwards:* a direction which it has not before been observed to have taken. It occurred to a surgeon, who fell from his horse with his left arm under his body. The swelling was so great when he was first examined that no dislocation could be detected. The only signs that indicated one were, that the left elbow was somewhat higher and more forward than the right, and that the motions of the arm backwards and outwards were limited and very painful. On the fifth day, the swelling being reduced, the head of the humerus was felt on a level with the acromion, and resting against its posterior margin. It was, with some difficulty, reduced by drawing the arm forwards and downwards; a distinct crack was heard, the patient himself seemed to feel the head of the bone pass into the glenoid cavity, and all the natural motions of the arm were restored, though they continued painful.

The only other accident that could have produced these symptoms is the dislocation of the long tendon of the biceps muscle; and, on the whole, we think it is more likely that this had occurred than that so strange a displacement of the humerus had taken place.

II. The chief end which Dr. Gobee has had in view in his work is, to give his cases—miscellaneous as they are—more value than those have which are published in an isolated form, by arranging them in their relation to atmospheric and other external circumstances. He has acted on the aphorism of Baglivi, " unicuique enim regioni sua est medicina, sua methodus," and has placed side by side with his tables of diseases occurring in each month—a complete meteorological table. In an hospital of less than 100 beds, it is of course impossible to draw from one year's experience any definite rules of atmospheric influence upon disease; but to those who are collecting statistics on the subject Dr. Gobee's work, from its completeness as far as it goes, and its accuracy, may be of great value. For the present we can only make the same use of it as of Dr. Kerst's.

Having described (p. 4,) a case of hemicrania, which was aggravated every night, and which he cured by sulphate of quina with opium, he says that this affords no proof that the pain was due to a latent intermittent fever; and adds, that for the last two years he has found the same treatment highly beneficial for the nocturnal pains of rheumatic ophthalmia. Whenever the eye has, with the exception of the redness and lachrymation, been pretty well through the day, and the boring pain comes on severely in the evening or at night, he orders, instead of local bleedings, two grains of quinine with a third of a grain of opium, to be taken every hour. The pain usually returns in but a slight degree next night, and the cure is sure to be completed by giving eight or ten grains of quinine, with a grain of opium on the next day.

Two cases of young soldiers are related (p. 83,) in which tartar emetic was used with evident benefit in well-marked delirium tremens, from hard drink. A grain was given every hour in water for about eight times. Stimulants were not administered.

The most singular fact in the book is the occurrence of four cases of acute inflammation of the spinal cord, two, at least, of which were so well marked that they deserve to be extracted. The first (p. 8,) occurred in a soldier, twenty-four years old, convalescent from slight inflammation of the fauces. One morning, having been as well as usual all the preceding day, Dr. Gobee found him with erysipelas of the nose and cheeks, and complaining of pain in the lower jaw, "but chiefly of a burning and quite intolerable pain in the loins. In examining the spine," says the author, "he shrieked out with pain when I came upon the first lumbar vertebra. The pain was fixed to one and the same place, and did not shift in the least. Before, during, and after the examination he had *shocks* which put the whole trunk in motion, returned every two or three minutes, and could only be compared with those from electricity or from strychnine." Sometimes the trunk only was thus affected. The patient's speech was difficult and indistinct, his tongue was dry, he had severe thirst, but could swallow without pain; his respiration was natural, his pulse small and a little accelerated. Dr. Gobee, diagnosticating *myelitis*, ordered him to be cupped largely over the loins, to be put directly afterwards in a warm-bath, and to take twelve grains of calomel in six doses at short intervals. These produced no relief, and shortly afterwards he was again cupped, with some advantage; but the shocks continued. In the afternoon, however, there was no amendment; the pain in the loins and sacrum was excessive, and the shocks continually recurred, but the mind was undisturbed. All that night was passed without sleep, but there was no delirium. In the morning the erysipelas had spread over the face. The shocks still continued, and he complained of pain when either the limbs or the body was touched. His general condition was the same. A grain of calomel having been given every hour during the night, small and frequent doses of tartar emetic were now administered, and he was put again in the warm bath. The latter produced relief of the pain and the shocks, but the erysipelas continued to spread, and he grew very weak. On the second night of his illness he was quiet and next day he had but little pain in the back, and the shocks were less frequent; touching the limbs was painful, and they were stiff and bent, so that the forearm could hardly be straightened. On the fourth day all had gone ill; he had been delirious and violent all night, he passed his urine and fæces involuntarily, and his pulse was very small. In the afternoon he was paraplegic, all night he was shrieking, and at noon next day he died. After death the cerebral and spinal dura mater was found very red, partly from the fulness of its own blood-vessels, and partly from the pia mater being seen through it.

"The injection of the latter was so great that over its whole surface, from the cerebrum to the extremity of the spinal marrow, not a spot could be found without excessively distended capillaries. Between the arachnoid and the brain and spinal marrow there was neither serum nor any morbid appearance. The colour and consistence of the surface of the brain were natural, so were the choroid plexuses, the corpora quadrigemina, and the thalami optici, but the corpora striata and the cerebellum were very soft. The medulla oblongata, as well as the cervical and dorsal portions of the cord were natural; but from the lowest dorsal vertebra to the cauda equina the softening was such that the spinal marrow, during examination, remained like pulp upon the fingers, and had lost all organic structure. Its colour was yellower at this than in the healthy part. No pus was to be found. All the other organs were healthy."

The second patient was a soldier, twenty years old, of weakly habit, who was admitted with severe pain in the head, especially in the supra-orbital region, which he ascribed to cold. There was no general disorder, and he was treated by purgatives, and, when these produced no good effect, by free local bleeding, warm baths, and tartar emetic: but they were all unavailing, he got no rest, and became melancholy. The pain in the right brow, and the snapping of the eye and intolerance of light which had speedily supervened on it, continued; but there was still no general disturbance. On the ninth night, however, there was a sudden change: he became delirious and violent, and passed his fæces involuntarily. In the morning he looked wild, and his pupils were dilated: his pulse was 120, and small; and his skin was dry. While in a warm bath, cold water was poured in a small stream upon his head for 15 minutes, and he was afterwards wrapped up very warmly. In the evening he was better, and he remained quiet all night. On the twelfth morning, however, he was talking to himself, continually, and he complained of severe pain when the eyelids were touched. He passed the day talking and singing, and in the evening a small quantity of morphia was given. It produced three hours' sleep, but he passed all the rest of the night in singing. Next day, the 14th of his illness, he was in a similar state, continually singing; but he now shrieked as if from pain, when his abdomen, and especially the inguinal region, was touched. He was now also much emaciated, though he had not been severely treated, his evacuations passed involuntarily, and his pulse was very small. From this state there was little change till he died; he had one short lucid interval, but he gradually sunk, and the excessive sensibility increased and extended, so that for two days before his death he shrieked when any part of the limbs or abdomen was touched. He died on the 17th day, and his body was examined thirty hours after,—the weather being very cold.

A large quantity of serous fluid was found between the membranes of the brain. The pia mater was very red and, at the ninth and tenth dorsal vertebræ, was covered with vascular patches. The gray substance of the brain was discoloured and yellowish: at the base there was a greenish transparent jelly-like false membrane in the tissue of the arachnoid. On the chiasma the arachnoid was very thick, and had on it a tuberculous body of the size of a pea which had probably pressed on the optic nerve and the first branch of the fifth pair. The surface of the hemispheres, the cerebellum, corpus callosum, fornix, corpora quadrigemina, thalami, corpora striata, and the spinal marrow, from the medulla oblongata to the cauda equina, were reduced to a pulpy consistence, so that the finger could be passed through them with scarcely the least resistance. The difference between the white and gray substances of the cord was also indiscernible, for it had all one whitish-yellow pulpy appearance. There were two ounces of bloody serum in the right lateral ventricle. The other organs were healthy.

The third case was less distinctly marked, and more complicated: the fourth was more chronic in its progress: but both tend to confirm the conclusions which the author draws from the first two; namely, that *shocks* of the trunk, like those produced by electricity, or by strychnine, are pathognomonic of the acute stage of inflammation of the cord, and that the exaggerated sensibility of the body, and the expression of pain, when any part of it is touched, appear to be as good indications of the softening having taken place.

The last fifty pages of Dr. Gobee's work are devoted to an essay on the ophthalmia of the Netherlands army, but this is too much a peculiar subject for consideration at present. The extracts which we have given include all that has particular interest; and as the authors of these works are both men of renown in their own land, they may serve to give some idea of the state and style of practice in Holland. It seems to be simple, guided by common sense, and plain, calm judgment, and is, if we may so speak, more *English* than that in any other country on the continent.

